# SAG4 DNA and Peptide Vaccination Provides Partial Protection against *T. gondii* Infection in BALB/c Mice

**DOI:** 10.3389/fmicb.2017.01733

**Published:** 2017-09-07

**Authors:** Jian Zhou, Lin Wang

**Affiliations:** ^1^Department of Orthopedics, The Second Xiangya Hospital, Central South University Changsha, China; ^2^Department of Electroneurophysiology, Jinan Children’s Hospital Jinan, China

**Keywords:** SAG4, toxoplasmosis, constructed plasmids, vaccine, *Toxoplasma gondii*

## Abstract

*Toxoplasma gondii* can lead to congenital infections in human. Surface antigen protein 4 (SAG4) of *T. gondii* is a potential stimulator for humoral and cellular immune responses. In the present study, a DNA vaccine encoding SAG4 from *T. gondii* was constructed and used to immunize BALB/c mice with peptide to evaluate the protective efficacy of the vaccine. The productions of IgG antibodies and cytokines (gamma interferon) from the vaccine (pSAG4/peptide) group were significantly higher than pSAG4 or peptide groups. After a lethal challenge by 1 × 10^4^ tachyzoites from the I strain (RH), the survival time of mice immunized by pSAG4/peptide was longer than that of pSAG4 or peptide immunized mice or control mice. Moreover, after challenging by 20 cysts of the II strain (PRU) of *T. gondii*, the number of brain cysts from pSAG4/peptide vaccinated mice was only 31% of the number in PBS injected mice. The findings suggested the SAG4 DNA vaccine with peptide led significant immune responses and improved the protection against *T. gondii* challenges.

## Introduction

The obligate intracellular pathogen *Toxoplasma gondii* (*T. gondii*) can infect a broad range of warm-blooded hosts including human beings and livestock (Montoya and Liesenfeld, 2004). In immunodeficient individuals, toxoplasmasis usually leads to severe consequences. *T. gondii* infection is the most common opportunistic infections and one of the main causes of death among AIDS patients. In addition, toxoplasmosis is the most common cause of central nervous system symptoms (Dubey et al., 2014). Additionally, women who acquire toxoplasmosis during pregnancy may lead to miscarriage and mental alteration (Bamba et al., 2014). Moreover, the collected results from epidemiologic survey indicated the high *T. gondii* prevalence can cause a large number of economic losses (Fajardo et al., 2013; Miao et al., 2013; Verin et al., 2013).

Although some chemical drugs (atovaquone, sulphadiazine, and pyrimethamine) could control *T. gondii* acute infections, they could not eliminate the chronic infection (Dupouy-Camet, 2004; Petersen, 2007). Reports indicated *T. gondii could* infect humans through the consumption of food contaminated by tissue cysts (Lu et al., 2017). Vaccines have been successful to provide protection against various infectious diseases including hepatitis B (Zhang N.Z. et al., 2013). Up to present, there is no vaccine suitable for protecting humans against *T. gondii* infection (Buxton and Innes, 1995; Kur et al., 2009). DNA vaccines that could induce long-lasting cellular and humoral immune responses were considered to be much safer than attenuated-live vaccines. DNA vaccines were considered to be effective ways to prevent the infection of *T. gondii* and had been studied in last several years. Previous studies indicated that ROP19 was a potent stimulator inducing humoral and cellular immune responses (Zhou et al., 2016b). Surface antigen protein 5D (SAG5D) has been considered as a promising candidate vaccine against toxoplasmosis (Lu et al., 2014).

*Toxoplasma gondii* surface proteins play a significant role in the process of recognizing host cells. Most of glycosylphosphatidylinositol (GPI)-anchored polypeptides related to target cell attachment were SAG1 family members (Lekutis et al., 2001). SAG4 protein was successfully detected on the surface of bradyzoites. Most infections with toxoplasmosis are chronic in the form of cysts. SAG4 protein expression is upregulated in bradyzoite, which prompted us to evaluate whether SAG4 could elicit effective immune responses against infection with a low virulence strain of *T. gondii* in the mouse model. According to previous study, SAG1 was believed to be the most promising vaccine candidate because it was regulated through humoral and cellular immune responses (Kasper and Khan, 1993; Cao et al., 2015; Han et al., 2017). In order to analyze the antigenicity and immunogenicity of SAG4, we used bioinformatics approaches to analyze and compare linear-B cell epitopes and Th-cell epitopes of SAG4 and SAG1. Moreover, the recombinant eukaryotic plasmid DNA vaccine pEGFP-C1-SAG4 (pSAG4) was constructed. Then we evaluated the ability of pSAG4 with peptide to protect mice from the invasion of virulent strain and attenuated strain of *T. gondii*.

## Materials and Methods

### Epitopes Prediction

Epitopes are the bases of protein antigenicity that determine antigen specificity (Van Regenmortel, 2009; Gao et al., 2012). SAG1, an excellent DNA vaccine that could induce effective cellular and humoral immune responses in immunized mice, is regarded as a promising vaccine condidate (Liu et al., 2006; Han et al., 2017). The PROTEAN subroutine of DNASTAR software (Madison, WI, United States) was used to compare the biochemical indexes between SAG4 and SAG1, including antigenic index, hydrophilicity plots, flexible regions, and surface probability. As shown in **Tables 1**, **2**, the peptides of SAG4 that had both higher score of linear-B cell epitopes and lower percentile of IC50 values than that in SAG1 were chosen. In addition, we used DNAMAN software to search for linear-B cell epitopes on SAG4 amino acid sequences.

**Table 1:** The linear-B cell epitopes in SAG1 and SAG4 amino acid sequences predicted by DNAMAN^a^.

Order	Amino acid position		Soore^b^	
		
	SAG1	SAG4	SAG1	SAG4
1	20–63	5–28	1.179	1.255
2	136–162	140–169	1.175	1.179
3	107–123	125–131	1.138	1.16
4	66–72	63–78^c^	1.127	1.148
5	197–206	84–96	1.109	1.136
6	211–223	111–119	1.103	1.121
7	244–250	50–57	1.063	1.052


*^a^The prediction was run for three times. Two or more amino acids condense into a peptide. ^b^Score interval (the lowest to the highest); high score = high binding. ^c^The used peptide 61–75 (SRPLEYIPPNPSQVL) was mostly embedded in the sequence 63–78.*

**Table 2:** IC50 values for SAG4 and SAG1 binding to MHC class II molecules obtained using the Immune Epitope Database^a^.

MHC II Allele^b^	Start-Stop^c^		Percentile Rank^d^	
		
	SAG1	SAG4	SAG1	SAG4
H2-IAb	26–40	61–75^e^	2.15	0.31
H2-IAd	21–35	42782	0.34	0.66
H2-IEd	14–28	15–29	18.45	3.61
HLA-DRB 1^∗^01:01	43095	43064	0.88	0.25


*^a^The Immune Epitope Database (http://tools.immuneepitope.org/mhcii). The prediction was run for three times. ^b^H2-IAb, H2-IAd, and H2-IEd alleles are mouse MHC class II molecules; the HLA-DRB1^∗^01:01 allele is a human MHC class II molecule. ^c^We chose 15 amino acids for analysis each time. ^d^Low percentile = high level binding, high percentile = low level binding. ^e^The sequence of the peptide (SRPLEYIPPNPSQVL) used in the study.*

To construct a positive vaccine against *T. gondii*, it is essential to explain which type of Th cell mediated immune responses. The Immune Epitope Database (IEDB)^1^ online service was used to analyze the half maximal inhibitory concentration (IC50) values of peptides that bind to the major histocompatibility complex (MHC) class II molecules of SAG4.

### The Mice and Parasites

Six- to eight-week-old female BALB/c mice were purchased from Shandong University Laboratory Animal Center (Shandong, China). They all were bred in groups of 10 per cage under specific-pathogen-free conditions and had free access to diet and tap water. All of the animal experiments were approved with the Ethics Committee of Medical School of Shandong University under Contract 2011-0015.

The low virulent strain (PRU strain) of *T. gondii* was maintained in our laboratory by passage of cysts in Kunming mice. The tachyzoites of *T. gondii* (RH strain) used in this study were harvested from human foreskin fibroblast cells. The *T. gondii* tachyzoites were used to create soluble tachyzoite antigens (STA) after washed by centrifugation and resuspended in sterile PBS. The parasite suspension was sonicated and centrifuged at 1000 × *g* for 20 min. Supernatant containing STAg was collected and kept at -70°C for further use (Zhang M. et al., 2013).

### Preparation of Plasmid

The entire coding sequences of the *T. gondii* SAG4 gene were amplified by PCR from genomic DNA of *T. gondii* strain RH with synthetic primers. *Trans Tag*^TM^ High FidelityDNA Polymerase (TransGen, Beijing, China) was used in PCR amplification.

SAG4: Forward primer: 5′-GGGGTACCATGACGAAAAATAAAATT-3′ Reverse primer: 5′-CGGGATCCTTACATTGATATCAACA-3′ (introduced *Kpn*I and *Bam*H I recognition sites, respectively, are underlined).

The PCR production of SAG4 gene was cloned into the pEASY-T1 Vector (TransGen, Beijing, China), digested with the appropriate restriction enzyme (*Kpn*I and *Bam*H I), and purified from agarose gels. SAG4 gene fragment was inserted into the eukaryocyte vector pEGFP-C1, generating pSAG4. The recombinant plasmids were then propagated in *Escherichia coli* DH5α and confirmed by restriction analysis and sequencing. Endotoxin-free plasmid DNA was isolated using a Plasmid Purification Kit (TianGen, Beijing, China). The concentrations of the purified plasmids were detected by spectrophotometer at 260 and 280 nm, and the 260:280 ultraviolet absorption ratio was between 1.8 and 2.0. All the plasmids were diluted into 1 mg/ml by sterile endotoxin-free PBS and stored at -20°C before use.

### Preparation of Polypeptide

In the present study, B-cell epitopes and Th-cell epitopes of SAG4 were analyzed using online service and software. Peptide sequence containing excellent B-cell epitopes and T-cell epitopes was chosen and synthesized. Peptide 61-75 (SRPLEYIPPNPSQVL) of SAG4 protein was selected and synthesized by Sheng Gong Biotechnology Company (Shanghai, China). The analytic High Performance Liquid Chromatography (HPLC) was used to purify the peptide.

### Expression of pSAG4 in HEK 293-T Cells

Human embryonic kidney (HEK) 293-T cells were grown at 37°C in a 5% CO_2_ incubator in Dulbecco’s Modified Eagle’s Medium (DMEM) supplemented with 5% fetal bovine serum, 100 mg/ml streptomycin, and 100 IU/ml penicillin. HEK 293-T cells were transfected with the eukaryotic expression plasmid (pSAG4) or an empty vector pEGFP-C1 (control plasmid) in liposomes (Lipofectamine^TM^ 2000). For the transfection, the cells were plated onto six-well tissue culture plates. After 24 h, the cells were transfected with pSAG4 in liposomes according to the manufacturer’s instructions. Lipofectamine^TM^ 2000 reagent was mixed with 1.0 μg plasmid DNA at a concentration of 10 μg/ml in DMEM, without fetal bovine serum and antibiotics, and incubated at room temperature for 20 min. The lipofectamine/DNA mixture was then overlaid on 75% confluent HEK 293-T cells, and the cells were incubated with the transfection mix for 6 h at 37°C and 5% CO_2_. At the end of the incubation, fresh medium was added and plates were returned to the incubator. Following 48 h of incubation, HEK 293-T cells were harvested. To detect the expression of pSAG4, total RNA was isolated from transfected HEK293-T cells using Trizol Up (TransGen, Beijing, China) and analyzed by reverse transcription (RT)-PCR with SAG4-specific primers. RT-PCR products were electrophoresed in an agarose gel, visualized by ethidium bromide staining, and confirmed by sequencing. The cells from different groups (pEGFP-C1, pSAG4, and control) were observed using fluorescence microscope under blue laser after incubation.

The SAG4 protein was examined by Western blotting. Specifically, cells were treated by RIPA Lysis Buffer (50 mM Tris pH 7.4, 150 mM NaCl, 1% Triton X-100, 1% sodium deoxycholate, 0.1% SDS) containing 1 mM protease inhibitor phenylmethanesulfonyl fluoride and centrifuged at 12,000 × *g* for 10 min. The supernatant was extracted and resuspended in 50 μl of SDS-PAGE buffer, boiled for 5 min, and 20 μl was loaded onto a 13% polyacrylamide gel. Proteins were transferred onto a polyvinylidene fluoride membrane via electrophoresis at 60 V for 3 h using a Bio-Rad Transfer System (Bio-Rad, Hercules, CA, United States). The membrane was saturated for 2 h with sealing fluid at room temperature and probed with an anti-*T. gondii* SAG4 antibody (goat) diluted 1:10,000 in saturation buffer. The membrane was incubated for 2 h with a horseradish peroxidase-labeled, rabbit anti-goat IgG antibody (Sigma) diluted 1:20,000 in saturation buffer, and signals were detected with the enhanced chemiluminescence system.

### DNA Immunization and *T. gondii* Challenge

Six- to eight-week-old female BALB/c mice were divided randomly into five groups (24 in each group). Three groups were immunized intramuscularly in their hind legs three times with 100 μl of PBS containing 100 μg pSAG4, 100 μg peptide, or 100 μg pSAG4/peptide on weeks 0, 2, and 4. The other two groups of mice were injected with empty vectors or with PBS as the control groups. Blood samples were collected from the caudal vein on days 13, 27, and 41 and the sera were stored at -20°C for enzyme-linked immunosorbent assays (ELISA). Two weeks after the final inoculation (day 42), spleens from four immunized mice of each group were collected under aseptic conditions. Half of the remaining mice were challenged intraperitoneally with 1 × 10^4^ tachyzoites of the high-virulence *T. gondii* RH strain, while 10 mice were infected intragastrically with 20 cysts of the *T. gondii* PRU strain. The mice challenged by the RH strain were observed and the number of survival days was recorded. Mice that showed signs of illness were sacrificed immediately using CO_2_ gas. After 1 month, brains were removed from mice infected with the PRU strain and homogenized in 1 ml PBS. The number of cysts in each brain was determined by counting three samples of 10 μl mixture per mouse.

### Antibody Detection

Blood samples were collected from the caudal vein on days 13, 27, and 42 and the sera were stored at -20°C for ELISA. *T. gondii*-specific serum IgG, IgG1, and IgG2a antibody levels were detected by ELISA. Briefly, 96-well plates were coated with 10 μg/ml sodium of STAg in 50 mM carbonate buffer (pH 9.6) (50 μl per well) at 4°C overnight. Plates were washed three times with PBS (pH 7.4) containing 0.05% Tween 20 (PBS-T20) and blocked with 1% bovine serum albumin for 2 h at room temperature. The mouse sera were diluted in PBS and incubated for 2 h. Plates were incubated with horseradish peroxidase-conjugated, goat anti-mouse IgG, IgG1, or IgG2a (Sigma) for 2 h. After washing, immune complexes were visualized by incubating with *o*-phenylenediamine dihydrochloride solution (Sigma) and 0.15% H_2_O_2_ for 30 min. The reaction was stopped by the addition of 2 M H_2_SO_4_. The optical density was then measured by an ELISA reader (BiotekELx800) at 490 nm. All assays were performed in triplicate.

### Cytokine Production

Cytokine levels were detected as described previously (Zhang M. et al., 2013). In this study, the levels of interleukin-4 (IL-4), interleukin-10 (IL-10), and gamma interferon (IFN-γ) from the experimental mice were detected.

### Statistical Analysis

SPSS 17.0 (IBM, Chicago, IL, United States) was used in the statistical analysis. Antibody levels and cytokine productions among the diverse groups were determined with a one-way analysis of variance. The survival time of mice was analyzed by the Kaplan–Meier method. The confidence interval data were used to analyze the survival time. Tukey’s studentized range test was used for post-test comparisons when a significant difference (*p* = 0.05) was observed among treatments. The difference was considered statistically significant if *p* < 0.05.

## Results

### Prediction of Epitopes

**FIGURE 1:**
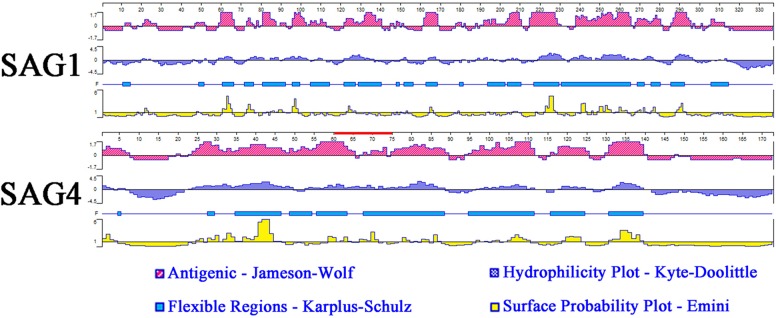
The linear-B cell epitopes of SAG1 and SAG4 predicted by DNASTAR in antigenic index, hydrophilicity plots, flexible regions, and surface probability rules. The red line on SAG4 was the position of peptide 61-75 (SRPLEYIPPNPSQVL) used in this study. The prediction was run for three times.

Analysis of amino acid hydrophilicity, the main power of protein folding, is the first step to understand protein folding that could be used for analysis of antigenic epitopes (accuracy rate: 56%) (Bai et al., 2012). In this study, DNASTAR software was used to analyze antigenic index, hydrophilicity plots, flexible regions, and surface probability of SAG4. SAG1 with excellent antigenic index and surface probability is an excellent vaccine candidate protein. The results of epitope analysis indicated SAG4 had a better antigenic index than SAG1. Additionally, the score of hydrophilicity plot and surface probability of SAG4 are significantly higher than SAG1 (**Figure 1**).

Furthermore, we used DNAMAN software to predict the linear-B cell epitopes on SAG4 amino acid sequence. The result of prediction indicated seven potential epitopes on SAG4 in **Table 1**. In addition, we used IEDB online service to analyze Th-cell epitopes of SAG4 and SAG1. The MHC class II molecules were related to half maximal inhibitory concentration (IC50) values from SGA4 peptides. As presented in **Table 2**, the minimum percentile ranks of SAG4 peptides were showed. The peptides of SAG4 that had both higher score of linear-B cell epitopes and lower percentile of IC50 values than that in SAG1 were chosen.

### Expression and Identification of Plasmids

**FIGURE 2:**
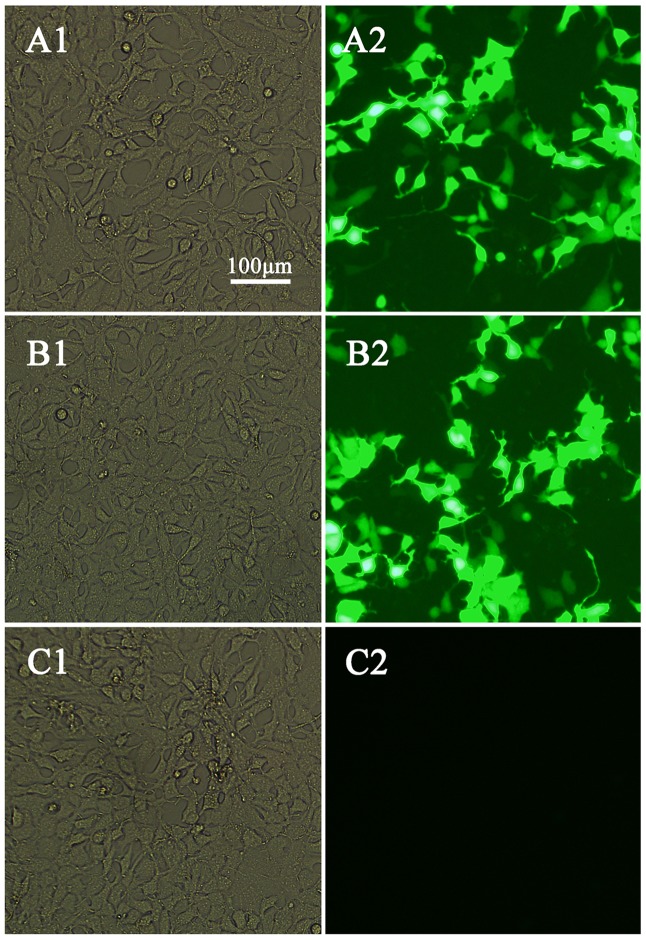
Direct immunofluorescence detection of the fusion protein in transfected HEK 293-T cells. **(A1)** Cells transfected with pEGFP-C1 detected under white light; **(A2)** cells transfected with pEGFP-C1 detected under blue light; **(B1)** cells transfected with pEGFP-C1-SAG4 detected under white light; **(B2)** cells transfected with pEGFP-C1-SAG4 detected under blue light; **(C1)** untransfected cells under white light; and **(C2)** untransfected cells under blue light.

**FIGURE 3:**
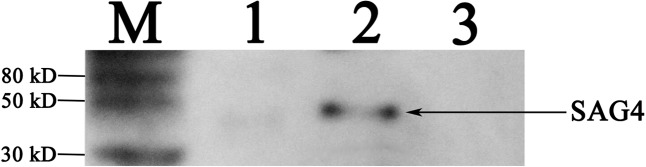
Detection of plasmid expression *in vitro* by Western blotting. M, protein marker; 1, HEK293T cells transfected with pEGFP-C1; 2, HEK293T cells transfected with pSAG4; and 3, untransfected cells.

We used polymerase chain reaction to detect SAG4 gene which then was ligated into pEGFP-C1 vector to construct pSAG4. The plasmids (pEGFP-C1 and pSAG4) were used to transfect HEK 293-T cells and cultivated for 48 h. Green fluorescence under exposure to blue laser was successfully found in pEGFP-C1- and pSAG4-transfected cells by fluorescence microscopy (**Figures 2A,B**), while no fluorescence was detected in control cells (**Figure 2C**). The expression of SAG4 gene [about 45 kDa, compound protein containing SAG4 protein (about 18.5 kDa) and GFP (about 27 kDa)] was detected in HEK293-T cells transfected by pSAG4 (**Figure 3**).

### Antibody Responses

**FIGURE 4:**
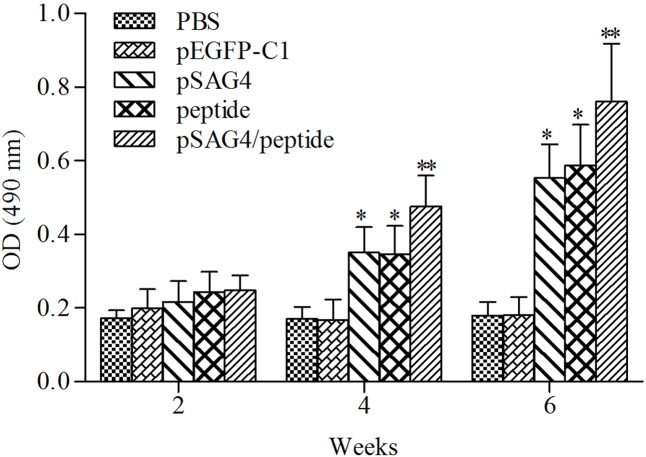
Measurement of specific IgG antibodies in immunized sera of mice. Twenty blood samples of each group were collected from caudal vein on days 13, 27, and 41. Results are expressed as means of the OD490 of the samples. *T. gondii*-specific IgG was used in the study. All the samples were run three times. ^∗^Compared with PBS or pEGFP-C1, *p* < 0.05 and ^∗∗^compared with pSAG4 or peptide, *p* < 0.05.

The collected serum of mice was detected using ELISA to examine the humoral immune response induced by pSAG4, peptide, or pSAG4/peptide. As presented in **Figure 4**, the levels of IgG antibodies from the experimental mice gradually increased following continuous immunity. Different antibody levels were detected from the sera of mice injected with pSAG4, peptide, or pSAG4/peptide compared to the mice immunized by PBS or pEGFP-C1 (*p* < 0.05), while no difference was found between PBS and pEGFP-C1 group. Furthermore, the levels of IgG antibodies from the pSAG4/peptide immunized mice were significantly higher compared with the pSAG4 or peptide injected mice (*p* < 0.05). The level of IgG antibody of the pSAG4-immunized mice was similar to that from peptide immunized mice (*p* > 0.05).

**FIGURE 5:**
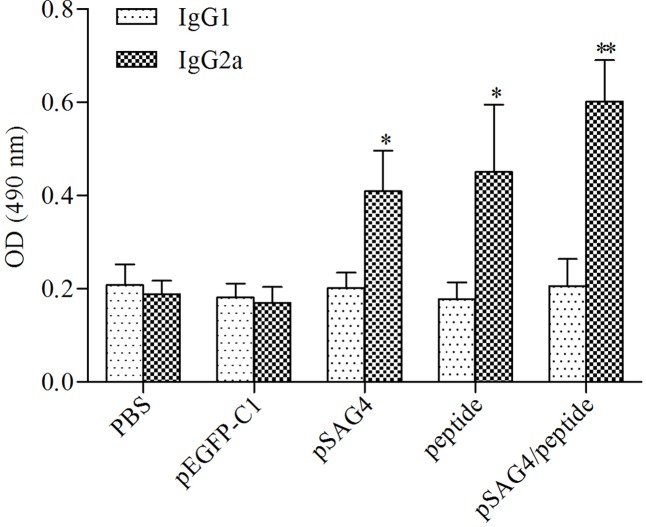
Determination of specific anti-*T. gondii* IgG1 and IgG2a in immunized mice. The sera of 20 mice of each group were collected from 2 weeks after the final immunization and the IgG subtypes levels were analyzed by ELISA. *T. gondii*-specific IgG1 and IgG2a were used in the detection. Results are expressed as means of the OD490 ± SD statistically significant differences (*p* < 0.05) compared to the control groups. Each sample was tested three times. ^∗^Compared with PBS or pEGFP-C1, *p* < 0.05 and ^∗∗^compared with pSAG4 or peptide, *p* < 0.05.

In order to examine the levels of subclass (IgG1 and IgG2a) of IgG, 2 weeks after the last injection, the serum of all related mice was prepared. As shown in **Figure 5**, a predominance of IgG2a over IgG1 was assessed in the mice injected using pSAG4, peptide, or pSAG4/peptide, while no difference was found in mice injected using PBS or pEGFP-C1. The foundings indicated that the plasmid mainly stimulated a Th1 type of immune response. Like the results of the levels of IgG antibodies, the level of IgG2a from mice vaccinated using pSAG4/peptide was close to twice that of mice injected by pSAG4 or peptide (*p* < 0.05). There was no difference of IgG2a level between the pSAG4-immunized mice and the peptide-immunized mice (*p* > 0.05).

### Cytokine Production

For the further study of the immune effect induced with the plasmid vaccine, the levels of cytokines (IFN-γ, IL-4, and IL-10) were detected using analyzing the culture supernatants of splenocytes in the injected mice. As presented in **Table 3**, the level of IFN-γ from mice immunized using pSAG4, peptide, or pSAG4/peptide was obviously higher compared to mice injected by PBS or pEGFP-C1 (*p* < 0.05). The mice vaccinated using pSAG4/peptide generated the highest IFN-γ (726.03 ± 103.98) level when compared to other groups. Although the pSAG4 (510.32 ± 71.97) immunized mice generated higher level of IFN-γ than the peptide (484.73 ± 99.57) immunized mice, no significant difference was found between them (*p* > 0.05). The levels of IL-4 in the splenocyte supernatants from the mice vaccinated using pSAG4/peptide (43.32 ± 7.61), pSAG4 (40.35 ± 6.94), and peptide (39.34 ± 7.5) were similar to those of mice injected by pEGFP-C1 (37.67 ± 9.27) and PBS (38.8 ± 7.44) (*p* > 0.05). Similar to the results of IgG isotype, the findings of cytokine detection indicated that the immunization of vaccines (pSAG4, peptide, and pSAG4/peptide) mainly induced a Th1-type immune response.

**Table 3:** Cytokine production by splenocyte^a^ cultures from immunized BALB/c mice.

Group	Cytokine production (pg/mL)^b^		
	
	IFN-y	IL-4	IL-10
PBS	55.95 ± 11.7	38.8 ± 7.44	39.51 ± 7.01
pEGFP-Cl	51.87 ± 11.6	37.67 ± 9.27	38 ± 8.15
pSAG4	510.32 ± 71.97^∗^	40.35 ± 6.94	41.66 ± 6.65
pepetide	484.73 ± 99.57^∗^	39.34 ± 7.5	37.44 ± 7.02
p S AG4/peptide	726.03 ± 103.98^∗^^#^	43.32 ± 7.61	40.58 ± 7.6


*^a^Splenocytes from four mice per group 2 weeks after the final immunization. ^b^Values for IFN-γ at 96 h, IL-4 at 24 h, IL-10 at 72 h are expressed as mean ± SD. ^∗^Compared with PBS or pEGFP-C1 group, *p* < 0.05. ^#^Compared with pSAG4 or peptide, *p* < 0.05.*

### *Toxoplasma gondii* Challenge Study

**FIGURE 6:**
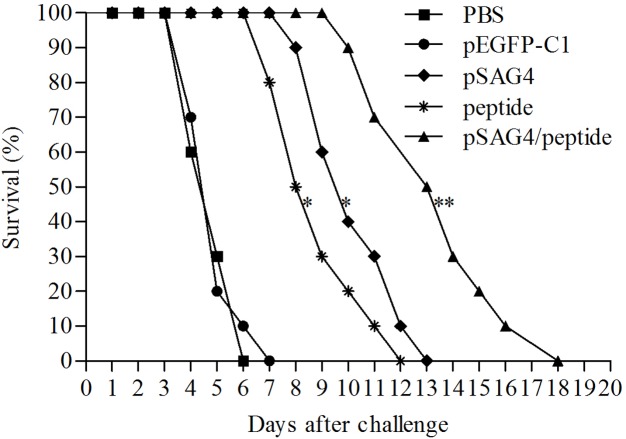
Survival curves of vaccinated BALB/c mice against *T. gondii* infection. The five groups of mice were challenged with 1 × 10^4^ tachyzoites of virulent *T. gondii* RH strain 2 weeks after the last immunization. Each group was composed of 10 mice and survival time was monitored daily for 17 days after challenge. ^∗^Compared with PBS or pEGFP-C1, *p* < 0.05 and ^∗∗^compared with pSAG4 or peptide, *p* < 0.05.

In order to evaluate the protection of the DNA vaccine, all the experimental mice were challenged using *T. gondii.* As shown in **Figure 6**, the mice of PBS mice and pEGFP-C1 mice died 4 days after virulent *T. gondii* infection (RH strain, 1 × 10^4^ tachyzoites per mouse), then all the mice were dead in 7 days. The survival time from the animal injected using pSAG4 (9.3 ± 1.64), peptide (7.9 ± 1.66), or pSAG4/peptide (12.5 ± 2.46) was longer than the mice vaccinated with PBS (3.9 ± 0.88) or pEGFP-C1 (4 ± 0.94) (*p* < 0.05). The mice of pSAG4/peptide group presented a longer survival time (18 days) than the pSAG4 or peptide vaccinated mice (*p* < 0.05). Moreover, the survival time of pSAG4-immunized mice was similar to the mice injected using peptide (*p* > 0.05).

In order to fully evaluate the protection of the DNA vaccine, 2 weeks after the last immunization, experimental mice were challenged intragastrically using 20 cysts of attenuated *T. gondii* strain (PRU). As **Table 4** showed, mice of pSAG4, peptide orpSAG4/peptide group had fewer brain cysts compared with the group injected by PBS or pEGFP-C1 (*p* < 0.05). The brain cysts of mice immunized using pSAG4/peptide were much lower than that of other mice (*p* < 0.05).

**Table 4:** The cysts in injected mice after challenge by cysts of PRU strain.

Challenged group^a^	Brain cysts per mouse (mean ± SD)^b^
PBS	1310 ± 246
pEGFP-Cl	1300 ± 271
pSAG4	865 ± 203^∗^
peptide	682 ± 182^∗^
pSAG4/peptide	406 ± 150^∗,#^


*^a^Ten mice from each group were challenged intragastrically by 20 cysts 2 weeks after the last immunization. All samples were run for four times. ^b^The mean number of cysts of each group (four mice) was obtained from every mice brain cysts in the group. ^∗^Compared with PBS or pEGFP-C1 group, *p* < 0.05. ^#^Compared with pSAG4 or peptide, *p* < 0.05.*

## Discussion

Bioinformatics has been widely used to predict gene structures, functions, and epitopes (Romano et al., 2011; Zhou et al., 2016a,c). The epitopes prediction plays a significant role in analysis for peptide immunogenicity and plasmid vaccine construction (Martin et al., 2004; Bai et al., 2012; Zhou et al., 2016b). In this work, several softwares and online services had been used to predict the B-cell epitopes and T-cell epitopes of SAG1 and SAG4. On the one hand, we had used DNASTAR and DNAMAN software to analyze the amino acid sequences of SAG1 and SAG4. The results showed that there were more antigenic index and surface probability regions of SAG4 than that of SAG1, which indicated that SAG4 protein had great potential to become an excellent B-cell antigen. On the other hand, the online IEDB database had been used to predict T-cell epitopes on SAG4 protein, and several great potential T-cell epitopes on the protein were selected. As presented in **Table 1**, a lower value of IC50 suggested a higher affinity, which demonstrated a better T-cell epitope. Obviously, SAG4 protein had much lower values of IC50 compared with SAG1, which indicated that this protein might be an excellent T-cell antigen. Overall, SAG4 had some excellent B-cell and T-cell epitopes, which indicated this protein had great potentiality to become a positive DNA vaccine against *T. gondii*.

Immunotherapy may be a promising approach to prevent toxoplasmosis. Prime-boost strategy was developed to strongly induce cellular immunity. Priming using DNA and boosting by polypeptide was considered to be a positive vaccine approach (Meng et al., 2013). Siachoque et al. (2006) used collected SAG1 peptides to vaccinate mice and got improving results. In another study, P30 and ROP18 peptides were successfully observed in human toxoplasmosis (Torres-Morales et al., 2014). SAG5A peptides, combining with DNA vaccine, were proven to be positive in protection against *T. gondii* in BALB/c mice (Torres-Morales et al., 2014). ROP38 may be a potential vaccine candidate against chronic infection of *T. gondii* (Xu et al., 2014).

Here, priming using DNA vaccine SAG4 and boosting by peptide (SRPLEYIPPNPSQVL) were performed. The BALB/c mice immunized intramuscularly using pSAG4, peptide, or pSAG4/peptide generated high levels of antibodies against *T. gondii* and stimulated protective immunity response. Furthermore, mice of pSAG4/peptide group stimulated both cellular and humoral immune responses, and could extend the survival days of the RH strain challenged mice and decrease the brain cysts in mice infected by *T. gondii* (PRU strain). Compared with previous studies, the vaccines in this study elicit a stronger protection against both RH strain and PRU strain of *T. gondii*. SAG4 DNA vaccine has the potential to be a successful DNA vaccine against *T. gondii*.

Recently, the importance of B-cell response against *T. gondii* infection has been discussed. The antibodies could restrain the attachment of tachyzoites to host cells to prevent spread of the parasite. The findings showed that levels of IgG antibodies from mice immunized using pSAG4/peptide were higher than other mice, which indicated that this vaccine could generate stronger humoral immunity response with the help of peptide.

Gamma interferon was considered to be important in resistance against *T. gondii* during both early and late phases of infection (Hiszczynska-Sawicka et al., 2010; Wu et al., 2012; Leroux et al., 2015). Furthermore, in the early phase of infection, the level of IL-4 can block the Th1 cell conversion and IFN-γ produce (Kang et al., 2000; Lekutis et al., 2001; Kim et al., 2007; Ahn et al., 2009; Goleva et al., 2009). In order to determine immune response polarization, the levels of IL-4 and IL-10 were detected using ELISA Kits. The pSAG4/peptide-injected mice could generate higher levels of IFN-γ and IgG2a compared to pSAG4, peptide, PBS, or pEGFP-C1 group, while the production of IL-4 was similar to each other. The results of this study showed that the cellular immune response from vaccinated mice tended to be a Th1 type. Splenocytes were considered to be a good marker for memory immune cells, especially in CD8 subsets of Th1 response, preventing the issues of low recovery for blood lymphocytes in mice models (Steinert et al., 2015).

The survival time in mice vaccinated with pSAG4, peptide, or pSAG4/peptide was longer than that of the mice injected by PBS or pEGFP-C1. Like Xu et al. (2014)’s report, after a lethal challenge with *T. gondii*, the mice immunized by single gene vaccines died in 12 days. Mice of pSAG4/peptide group obtained 18 days survival time after a challenge of *T. gondii*. Furthermore, compared to the mice of control groups, the group vaccinated by pSAG4, peptide, or pSAG4/peptide had a significant reduction of brain cysts. The number of brain cysts in group immunized using pSAG4/peptide was only 31% of the control group, which was similar to the report of Lu et al. (2015). These results suggested that the DNA vaccine encoding SAG4 was able to bring partial protection in BALB/c mice against *T. gondii* (RH and PRU strain) infection. This study provided a basis of the construction of a DNA vaccine against virulent and low virulent *T. gondii*.

In this report, we used bioinformatics approaches to analyze B-cell epitopes and T-cell epitopes on SAG4 protein. Moreover, DNA vaccine and peptide were injected into mice to assess the immune protection stimulated with the vaccines. The mice injected with the DNA vaccine stimulated positive humoral and cellular immune responses. The DNA vaccine (pSAG4) had been proven to effectively reduce the number of cysts in brains of the immunized mice and positively prolong the survival time of vaccinated mice after a challenge of *T. gondii*, especially with the help of peptide. SAG4 DNA vaccine has the potential to be a successful DNA vaccine for *T. gondii*. The present study provided the foundation for further studies of multiple strain-resistant DNA vaccine of *T. gondii*.

## Ethics Statement

This study was approved by the Institutional Animal Care and Use Committee of Shandong University under Contract 2011-0015, and the animals were kept and the experiments were performed in accordance with committee’s criteria for the care and use of laboratory animals. All mice were maintained in specific pathogen-free conditions, and all efforts were made to minimize suffering. Humane endpoints to reduce pain or distress in mice were used via euthanasia. Mice were sacrificed immediately using CO_2_ gas before the brains were removed. Generally, mice were placed in a chamber and CO_2_ was administered at a concentration of 60–70% over a 5-min exposure time, after which the cervical dislocation method was sometimes used to ensure that effective euthanasia had occurred.

## Author Contributions

LW conceived and designed the study and JZ carried out the experiments and drafted the manuscript.

## Conflict of Interest Statement

The authors declare that the research was conducted in the absence of any commercial or financial relationships that could be construed as a potential conflict of interest.

## Footnotes

1 http://tools.immuneepitope.org/mhcii
